# The moderating role of sociodemographic and work-related variables in burnout and mental health levels of Mexican medical residents

**DOI:** 10.1371/journal.pone.0274322

**Published:** 2022-09-16

**Authors:** Alejandra del Carmen Dominguez-Espinosa, Sandra Irma Montes de Oca-Mayagoitia, Ana Paola Sáez-Jiménez, Javier de la Fuente-Zepeda, Lilia Monroy Ramírez de Arellano

**Affiliations:** 1 Psychology Department, Universidad Iberoamericana, Mexico City, Mexico; 2 SEDESA, Dirección de Formación, Actualización Médica e Investigación, Secretaria de Salud, Mexico City, Mexico; Imam Abdulrahman Bin Faisal University, SAUDI ARABIA

## Abstract

**Objective:**

To explore the moderating effects of sociodemographic and work-related variables on levels of burnout and mental health among medical residents.

**Method:**

A cross-sectional online survey was administered at the beginning of the second wave of COVID-19 at different public teaching hospitals where medical residents practiced in Mexico City. A total of 201 medical residents of different years completed the survey.

**Results:**

Different univariate inferential analyses on the level of burnout and mental health indices showed significant differences between sex, marital status, previous reports of physical illness or psychological conditions, and residency ranking. However, the effect sizes of those differences were of low to medium size. A predictive path analysis revealed that the three stages of burnout (emotional exhaustion, depersonalization, and achievement dissatisfaction) negatively affect psychological wellbeing and positively affect psychological distress. Finally, even though sociodemographic variables showed some significant variation, the effect sizes were small and did not moderate the direct effect of burnout on mental health indices.

**Conclusions:**

Medical residents deling with every day medical situations, will be exposed to stressors that might increase the probability to experience emotional exhaustion. This would negatively affect levels of wellbeing and positively affect distress, despite their sociodemographic characteristics.

## Introduction

There is no doubt that the most affected group of professionals during the COVID-19 pandemic is that of health care providers, particularly in the medical sector [1, 2]. Among them, there is a specific vulnerable group that includes medical students, interns, and residents. According to different studies across the world during the last decade, medical students, interns, and residents are among the most stressed students with medium to high levels of burnout [3–8] and psychological distress, depression and anxiety [9, 10]. Burnout and psychological distress are not only personal concerns but also institutional concerns, as they affect the work environment where students and residents are enrolled [11]. Despite the enormous amount of information regarding burnout and mental health alterations in medical students and residents, there is still room to explore for stronger evidence of the susceptibility of residents according to different socioeconomical and work-related variables, as the results are not convincing in many cases. For this reason, the main objective of the present paper was to investigate the impact of different sociodemographic variables, including residency ranking and the prevalence of previous physical and psychological illnesses, on burnout levels and mental health indicators in Mexican medical residents. Identifying the influence of sociodemographic variables as moderators, will help to tailor psycho-educational intervention for the medical residents, therefore, making future interventions more effective.

### Burnout in medical residents

The Interinstitutional Commission for the Training of Human Resources for Health [12], through the Committee on Graduate Teaching and Continuing Education (Comité de Enseñanza de Posgrado y Educación Continua), oversees organizing and supervising all postgraduate medical programs and guaranteeing that all students enrolled in any postgraduate medical program in Mexico act as trainee medical doctors under the direct supervision of professors or adjunct professors. In general, the regular term for a postgraduate program in medicine can vary from three to five years, and students are required to do planned on-call hours in line with their specializations [13].

According to a series of studies worldwide, medical students and residents are nearly 2.5 times more susceptible to experiencing higher levels of burnout than practitioner physicians, particularly in the first two dimensions of the process model of burnout [14–18]. The process model of burnout consists of three stages: 1) Emotional exhaustion, which is the response to job demands that drain emotional resources; 2) Depersonalization, consisting of defensive coping characterized by cynicism; and 3) Reduced personal accomplishment—or achievement dissatisfaction, characterized by a sense of inefficacy and failure [19–23].

Some factors that have been suggested as boosters for burnout among medical residents is the duty to be on-call for 24-hour periods, causing sleep deprivation and, in consequence, increasing the number of omissions and errors [24, 25], with an additional concern of not having enough resources to attend to patients [26].

Other variables that have been explored in relation to burnout levels include sex [27], having a chronic disease, [28], being single [29], residency ranking and medical field [30–38], work overload [39–42], having higher scores in neuroticism [33, 43, 44], not receiving proper recognition from supervisors [29] or being humiliated by supervisors or other higher-ranking physicians [45].

Additional stressors during the COVID-19 pandemic are the inability to obtain protective equipment during routine clinical work [46, 47] and the fear of infecting a loved one or being infected with COVID-19 [48–50]. However, the empirical evidence is not conclusive; as in some cases, these factors, including the number of working hours [51] or years of practice [52], did not show size effects similar to previous findings or a significant effect [39, 53–55].

### Mental health indicators

Medical students and residents are prone to experience stress, depression, and psychological distress, with a prevalence as high as 90% of the cases studied globally [9, 56]. This prevalence can be moderated by different factors related to years of study. El-masry et al. [38] found that senior students are more concerned with the fear of harming patients or defective clinical practice skills, while first-year students are more concerned about getting infected or working in limited training conditions[26]. Similarly, Iorga et al. [57] identified that first-year students experience more depressive symptoms than senior students, but the latter experience more stress. Additionally, being a first-year resident increases the likelihood of experiencing higher levels of burnout [58, 59], mainly among surgical/emergency residents [60].

The hypothesis behind the difference between residency ranking, that is, in the year of study in which the medical residents are, and the perception of lowest ranking status (1^st^ years) is supported by the junior residents category when compared to the rest of the residents attending the same academic program or at the same hospital; junior residents received less support, perceived ambiguity in the roles to be played, were sometimes threatened by senior residents or supervisors [12, 45], and had lower levels of social skills and clinical experience [61, 62]. However, other findings suggest that second-year students are more susceptible to burnout than first-year students [34, 63], probably because second-year students are no longer perceived as junior doctors and their responsibilities increase.

Analogous to these previous findings, Alkhamees et al. [64] found that 16% of medical residents during the COVID-19 pandemic presented higher levels of burnout and depressive symptomatology, and this is especially important in the first two years of training. Additionally, being positive or symptomatic for COVID-19 increased levels of depression and stress [65].

### Resilience in residency environment

According to Abedini et al. [4], resolving workplace challenges, nurturing personal lives and taking time off can reduce the levels of burnout among medical residents, and wellness programs and counseling sessions can help to overcome burnout [66]. Additionally, to minimize the impact of burnout, program directors and authorities should limit tasks and team-related work changes whenever possible [67]. In terms of academic achievement, there have been efforts to make the best of the pandemic, and teaching and learning accommodations have proven to be fruitful [68]. However, if we truly want to reduce stressors in the work environment and create a healthier atmosphere for medical residents by means of individual-focused and organizational strategies [69], it is important to explore the moderating role of sociodemographic and work-related factors that have proven to regulate, to some extent, the prevalence of burnout and mental health indicators among medical residents.

## Method

This study was part of a psychoeducational intervention program aimed at supporting medical residents during the second wave of the Covid-19 pandemic. The data presented here only corresponds to the screening test that consisted of a cross-sectional online survey to assess the levels of burnout and mental health in medical residents. The results presented here were used to develop resident-focused infographics, based on the practical implications reported in this article.

The present research consisted of a in a risk-free, nonintrusive way, as the participant were asked to give their consent before endorsing the screening test, voluntarily and anonymously. The sample was collected during the second wave of Covid-19 infections in Mexico City.

The Research Ethics Councils from SEDESA and from the Iberoamerican University gave their approval for the present research. Registration number: CEI-NC/101-010-030-20.

### Participants

An initial sample of 229 medical residents participated in the study during the fall semester from November 9^th^, 2020 to February 11^th^, 2021. All residents were rotating in public hospitals affiliated with the Mexico City Health Ministry (Secretaría de Salud [SEDESA]). After excluding 28 cases due to missing data, a valid sample size of 201 participants, aged between 25 and 41 years old (M_age_ = 29.93 years, SD_age_ = 2.45 years), was used for the analyses. Of these 201 participants, 63% were female; 77% were single and, according to residency ranking, 25% were 1st-year, 30% were 2nd-year, 27% were 3rd-year and 9% were 4th and 5th-year residents (S1 Table).

### Measures

The survey consisted of a sociodemographic section in which participants were asked to indicate their sex, age, marital status, parental status, residency ranking, type of on-call responsibilities, numbers of days off, hospital affiliation, medical specialty, the number of estimated patients seen per week, and if they were suffering from a chronic disease or psychological affliction. Immediately thereafter, the participants were presented the following psychological scales in the following order and with six discrete response options.

The Mental Health Index (MHI) - 38 item version [70]. As its name suggests, the MHI consists of 38 items that, depending on different coding combinations, permits obtaining a global mental health index, two general subindices and six specific subindicators. The MHI was constructed based on the bi-dimensional model of mental health that include positive and negative items and address psychological distress and well-being as higher order orthogonal multidimensional factor. The 38 items followed a hierarchical structure on the results of exploratory and confirmatory factor analyses that suggested a model composed of a general underlying higher order structure of two correlated factor -psychological distress and well-being- with five correlated lower order factor- depression, anxiety, emotional ties, general positive affect, and loss of behavioral emotional control- and a single item of life satisfaction. Each index is calculated based on the sum of the different items: satisfaction with life (1 item), emotional ties (2 items), positive affect (10 items), anxiety (9 items), depression (4 items), and loss of behavioral/emotional control (9 items); the first three subindicators correspond to the wellbeing subindex (13 items plus one extra item); the final three subindicators correspond to the distress subindex (22 items plus two extra items). Finally, the MHI score is obtained by adding the 38 total indicators of the inventory. Each item is presented in a frequency format over the last month.

The Burnout Index, EDO—23 item version [22, 23, 71]. The current EDO is a Likert-type summative scale that measures three dimensions of burnout after Maslach and Jackson’s process model [19]: emotional exhaustion (9 items, e.g., I have a hard time getting up in the morning to go to work), depersonalization (9 items, e.g., I have found that the people I serve respect me more if I treat them badly) and achievement dissatisfaction (5 items, e.g., My work activities no longer seem important to me). A global index of occupational burnout can be obtained by summarizing all 23 items.

### Materials and procedure

The invitation to participate in the study was sent to all medical residents rotating in public hospitals affiliated with the Mexico City State Health Ministry (Secretaría de Salud, SEDESA[Acronym in Spanish]) by the time of the present study. Through the teaching units of each hospital, the survey link was sent to the medical residents using the SurveyMonkey online tool [72]. Residents were invited to collaborate in this research and were asked to give their informed consent to ensure their voluntary and anonymous participation. The participants did not receive any financial compensation for answering the survey or any sanction for their absence from participating. The average time to complete the survey was 20 minutes.

### Statistical analyses

Descriptive analyses (means, standard deviations, skewness, and kurtosis, bivariate correlations (Person formula), and mean contrasts (t-test and One-Way Analysis of Variance [ANOVA’s] with post-hoc Scheffé test) were performed over the complete data matrix to identify potential moderators of the effect of burnout on mental health scores. A path analysis and a series of multigroup comparisons were used to test for moderation effects by means of z-scores on critical ratio on regression weights. All statistical analyses were run using IBM SPSS® [73], the path analysis was estimated using the IBM AMOS® [74] and the contrast for the moderators using the free software Stats Tools Package [75].

## Results

### Descriptive analyses

Before running the analysis, we run quality checks for outliers and missing data. Neither the missing data nor the number of outliers (n = 6 total) were of concern for further analysis. Table 1 shows that the distribution of the metric variables in the total sample is close to symmetric, as the skew and kurtosis information is not above the cut point criterion of 2. The highest mean score was in emotional ties, and the lowest was in achievement dissatisfaction. The minimum reliability index is .75, which suggests that all metric scores are internally consistent and hence reliable.

**Table 1:** Descriptive information for the metric psychological variables.

			*n*	*M*	*SD*	α	Range	Skew
Mental Health Index			195	4.00	0.95	.97	1.61–5.87	-0.15
	Psychological wellbeing		200	3.85	0.86	.89	1.86–5.64	0.06
		General positive affect	201	3.78	1.02	.93	1.50–6.00	0.10
		Emotional ties	200	4.18	1.38	.83	1.50–6.00	-0.26
		Life satisfaction	201	4.13	0.91	-	3.00–6.00	0.26
	Psychological distress		195	2.81	0.97	.96	1.08–5.33	0.41
		Anxiety	200	3.07	1.08	.93	1.00–5.67	0.25
		Depression	199	2.74	1.09	.89	1.00–5.75	0.49
		Loss of B/E control	198	2.37	0.98	.92	1.00–5.11	0.67
Burnout Index			191	2.69	0.80	.87	1.22–4.70	0.39
		Emotional exhaustion	196	3.69	1.10	.75	1.00–600	0.13
		Depersonalization	194	2.07	0.86	.81	1.00–4.44	0.80
		Achievement dissatisfaction	195	1.95	1.22	.90	1.00–6.00	1.32

*Note*: α indicates the reliability index by means of Cronbach’s alpha. Life satisfaction consists of only one indicator. B/E = Behavioral/Emotional

### Bivariate correlations

As expected, the correlations between burnout and mental health indicators varied from moderate to high effects. In Table 2, the largest effect size is between the global mental health index and the total burnout score [r^2^ = -.80, *p* < .01], in the expected direction.

**Table 2:** Correlations between mental health indicators and burnout dimensions.

			Burnout index	Emotional exhaustion	Depersonalization	Achievement dissatisfaction
Mental Health Index			-.80	-.77	-.44	-.62
	Psychological wellbeing		-.77	-.71	-.45	-.61
		General positive affect	-.77	-.74	-.42	-.59
		Emotional ties	-.61	-.51	-.43	-.49
		Life satisfaction	-.51	-.54	-.20	-.43
	Psychological distress		.78	.75	.42	.60
		Anxiety	.69	.72	.32	.50
		Depression	.74	.73	.39	.57
		Loss of B/E control	.76	.69	.43	.62

Note: Analyses were performed with Pearson’s r formula. All correlations were significantly different from zero at *p* < .01.

### Group contrasts

A series of group mean comparisons were conducted using almost all demographic information with t-tests and ANOVA contrasts. The first contrast was on sex, and the results showed that men obtained, on average, higher and significantly different scores than women in positive affect [*t* (198) = 2.15, *p* = .03, *d* = .31], satisfaction with life [*t* (135) = 2.08, *p* = .04, *d* = .31], and depersonalization [t (118) = 2.16, p = .03, d = .34]. In the cases of depression [*t* (196) = -2.13, *p* = .03, *d* = .32] and exhaustion [*t* (193) = -2.68, *p* = .01, *d* = .41], women scored higher and significantly different than men did, although the effect sizes for these comparisons were small in magnitude (S2 Table).

To perform a group comparison between the different marital statuses, it was necessary to merge divorced, widowed, and separated participants into the single category, as these three groups accounted for less than 4 cases. The married group included partnered participants (19 cases). The single group scored lower in the MHI and wellbeing indicators and higher in the distress indicators. Of these significant results, the effect size was of medium scope (range .43 to .57). There were no significant results between the two groups in the burnout index or in its subscales. (S3 Table).

To run the comparative analyses between residents with or without any reported diseases or conditions, the groups were first segmented by those that indicated some physical or psychological condition. Physical illness included any chronic metabolic diseases (e.g., diabetes, hypertension), and psychological conditions included any affective or emotional disorders (e.g., anxiety, depression). Almost all contrasts for the mental health variables were significantly different from zero and in the expected direction; those with no physical or psychological conditions scored higher in the wellbeing subindicator and lower in the distress subindicator, burnout index and emotional exhaustion. No significant effects were obtained only for depersonalization [*t* (192) = -0.93, *p =* .354] and achievement dissatisfaction [*t* (193) = -1.98, *p* = .094] (S4 Table).

To explore the contrasts by residency ranking, the highest residency rankings of 4^th^ (28 cases) and 5^th^ (9 cases) years were considered as one group. By means of a series of one-way ANOVA’s and Scheffé post hoc analyses, significant differences between first-year and second-year residents in the MHI [:\*F*(3, 196) = 2.84, *p* = .04] and life satisfaction scores [*F*(3, 196) = 3.54, *p* = .01] were observed, where the first-year group scored lower; the first-year group scored higher in distress [*F*(3, 196) = 3.06, *p* = .03], anxiety [*F*(3, 196) = 2.67, *p* = .05], depression [*F*(3, 196) = 2.64, *p* = .05], loss of behavioral/emotional control [*F*(3, 196) = 2.96, *p* = .03], and emotional exhaustion [*F*(3, 196) = 3.05, *p* = .03] in general, according to the η^2^, small size effects (S5 Table).

A series of additional contrasts were performed by educational level, type of on-calls, number of days off, and parenting status, and no significant results were obtained; therefore, no tables are shown.

### Moderation effects

**Fig 1:**
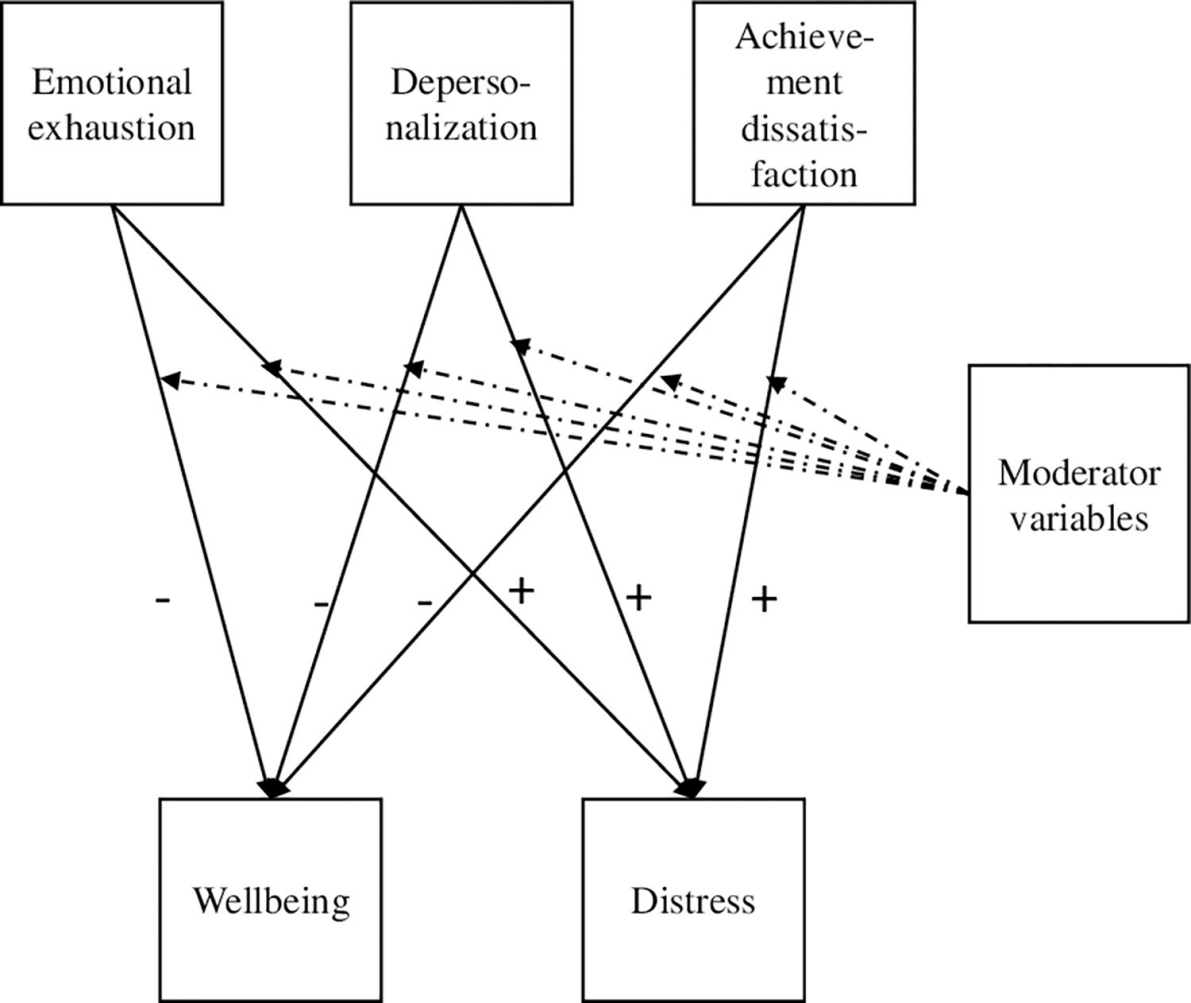
Conceptual model of the path analysis with moderator effects. Measurement error terms and covariances are not shown.

A path analysis was performed to evaluate the direct effects of the three burnout dimensions on the wellbeing and distress subindices (Fig 1). The results are shown in Table 3. As expected, the sign of the direct effects is negative for wellbeing and positive for psychological distress. The larger effects are for the prediction of emotional exhaustion for both mental health subindices.

**Table 3:** Path analysis regression weights of burnout on mental health subindices.

		B	SE B	β	*p*
Psychological wellbeing					
	Emotional exhaustion	-0.43	0.04	-0.54	< .001
	Depersonalization	-0.19	0.06	0.19	< .001
	Achievement dissatisfaction	-0.18	0.04	-0.25	< .001
Psychological distress					
	Emotional exhaustion	0.53	0.04	0.60	< .001
	Depersonalization	0.18	0.05	0.16	< .001
	Achievement dissatisfaction	0.18	0.04	0.23	< .001

*Note*: N = 185 due to listwise deletion.

To prove the moderation effects of sex, marital status, presence of any physical or psychological conditions, and between 1^st^ and 2^nd^ and 1^st^ and 3^rd^ year residents, a series of multigroup contrasts were performed. The results are shown in Table 4. None of the z-scores pairwise comparisons on the critical ratios from the regression estimates [75] were large enough to reject the null hypotheses, with the implication of a significant moderator effect affecting the relations between burnout dimensions and wellbeing and distress scores. In other words, even if there were slightly different estimates for each group, the effects were not large enough to make a different prediction for either group.

**Table 4:** Pairwise contrast on the estimates of the sociodemographic variables for the path analysis.

			Male		Female		
			**Estimates**	***p***	**Estimates**	***p***	**z-score**
Wellbeing	<---	Emotional exhaustion	-0.457	.001	-0.390	.001	0.743
	<---	Depersonalization	-0.184	.015	-0.245	.001	-0.575
	<---	Achievement dissatisfaction	-0.158	.023	-0.179	.001	-0.236
Distress	<---	Depersonalization	0.105	.203	0.254	.001	1.316
	<---	Emotional exhaustion	0.515	.001	0.494	.001	-0.220
	<---	Achievement dissatisfaction	0.159	.035	0.220	.001	0.643
			Single		Married		
			**Estimates**	***p***	**Estimates**	***p***	**z-score**
Wellbeing	<---	Emotional exhaustion	-0.417	.001	-0.423	.001	-0.053
	<---	Depersonalization	-0.184	.001	-0.269	.046	-0.587
	<---	Achievement dissatisfaction	-0.160	.001	-0.166	.089	-0.050
Distress	<---	Depersonalization	0.182	.004	0.079	.471	-0.811
	<---	Emotional exhaustion	0.518	.001	0.491	.001	-0.283
	<---	Achievement dissatisfaction	0.187	.001	0.215	.007	0.294
			**w/o Illness**		**w/Illness**		
			**Estimates**	***p***	**Estimates**	***p***	**z-score**
Wellbeing	<---	Emotional exhaustion	-0.415	.001	-0.408	.001	0.080
	<---	Depersonalization	-0.227	.001	-0.129	.062	1.006
	<---	Achievement dissatisfaction	-0.186	.001	-0.112	.043	0.947
Distress	<---	Depersonalization	0.185	.010	0.136	.093	-0.445
	<---	Emotional exhaustion	0.484	.001	0.569	.001	0.886
	<---	Achievement dissatisfaction	0.178	.002	0.190	.004	0.134
			**1** ^**st**^ **-year**		**2** ^**nd**^ **-year**		
			**Estimates**	***p***	**Estimates**	***p***	**z-score**
Wellbeing	<---	Emotional exhaustion	-0.480	.001	-0.566	.001	-0.851
	<---	Depersonalization	-0.322	.001	-0.288	.005	0.240
	<---	Achievement dissatisfaction	-0.091	.223	-0.047	.528	0.421
Distress	<---	Depersonalization	0.285	.019	0.205	.051	-0.502
	<---	Emotional exhaustion	0.548	.001	0.583	.001	0.308
	<---	Achievement dissatisfaction	0.248	.005	0.097	.204	-1.304
			**1** ^**st**^ **-year**		**3** ^**rd**^ **-year**		
			**Estimates**	***p***	**Estimates**	***p***	**z-score**
Wellbeing	<---	Emotional exhaustion	-0.480	.001	-0.348	.001	1.127
	<---	Depersonalization	-0.322	.002	-0.305	.001	0.123
	<---	Achievement dissatisfaction	-0.091	.223	-0.204	.022	-0.972
Distress	<---	Depersonalization	0.285	.019	0.159	.161	-0.762
	<---	Emotional exhaustion	0.548	.001	0.463	.001	-0.614
	<---	Achievement dissatisfaction	0.248	.005	0.193	.073	-0.395

## Discussion

Even before the COVID-19 outbreak, medical students and residents were vulnerable groups that consistently experienced burnout and mental health issues [3, 4, 6, 9, 15, 38, 56, 57]. Likewise, as the COVID-19 pandemic continues, levels of burnout and emotional distress among medical residents across the globe continue and intensify due to additional stressors [2, 18, 28, 30, 46, 48, 50, 58, 64, 68].

Based on previous findings, we explored the potential moderating effect of different sociodemographic and work-related variables, and surprisingly, none had a significant effect between the relation of burnout and mental health; even variables at the univariate level showed some significant differences. These results are in line with previous findings where the differences in separate samples were not conclusive [36, 39, 51–55]. The role of sex, marital status, and suffering from a physical illness showed significant differences between groups in some of the indices; however, the effect sizes of those differences were not large enough to compromise the prediction.

A particular interest was in testing whether residency ranking affected the level of burnout or mental health indices, as previous researchers suggested [12, 34, 45, 61–63]; nevertheless, there was no sufficient empirical support to reject that suggestion. Despite the observed significant difference between 1^st-^, 2^nd-^ and 3^rd^-year residents on the univariate analyses, the differences did not prove to be substantial in affecting the direct paths of the predictive model.

The findings of the present research suggest that medical residents, rotating in the public system or dealing with every day medical situations, will be exposed to stressors that might increase the probability to experience emotional exhaustion. This would negatively affect levels of wellbeing and positively affect distress, despite sex, marital status, residency ranking, or having any physical or psychological concerns.

### Practical recommendations

In accordance with scientific literature, it is possible to partially reverse the situation of occupational burnout of medical residents through individual interventions that provide self-care tools, as well as through organizational interventions that promote a better climate and working conditions [69]. The recognition that occupational burnout is not an exclusive problem of the individual, but also concerns training and work organizations is a starting point [11].

The present research highlighted three main problems among medical residents: 1) Increased levels of exhaustion, 2) Increased levels of anxiety and 3) Lower levels of positive affect.

Emotional exhaustion refers to the progressive loss of energy, tiredness, enervation, and fatigue; situations in which workers feel that they can no longer give more of themselves on an affective level [19, 20, 23]. In this sense, medical residents, working in contact with patients daily, makes their emotional resources been demanded continuously, therefore, experience emotional exhaustion. To help medical residents to overcome emotional exhaustion, we recommend using strategies focus on collaborative problem solving. It is necessary to create a set of good practices that foster work teams focus on identify and solve the most common problems among the students and give some feedback to those having troubles to solve everyday problems. The promotion of a positive work environment would increase if hospital authorities can guarantee adequate physical spaces where medical residents can rest or mental disconnect from work tasks and scheduled structured leisure spaces.

Anxiety is an emotional response that arises when people are exposed to danger or threat. Muscle relaxation, diaphragmatic breathing, and mindfulness techniques are effective low-cost scientific evidence-based interventions to deal with anxiety levels [76, 77]. Promoting these interventions among the medical residents can be a strategy to decrease non-pathological anxiety levels [78]. A major institutional commitment is needed to provide access to mental health services to medical residents as a part of a long-lasting intervention [3]. Access to psychotherapy and crisis intervention sessions, workshops, and psychoeducational activities with a focus on cognitive restructuring, aim to replace negative thoughts and promote self-care, will prevent, and reduce anxiety.

Positive affect refers to pleasurable emotionality manifested through motivation, energy, desire for affiliation, and feelings of mastery, achievement, or success. People with high affection tend to experience feelings of satisfaction, pleasure, positive enthusiasm, energy, friendship, union, sustained and trust [4, 79]. In our findings, the levels of positive affect were the lowest, therefore, we recommend promoting gratefulness, appreciation, and self-recognition among colleagues and classmates [80, 81]. More specifically, to highlight welldoings instead of wrongdoings; emphasize positive feedback instead of punitive teaching practices.

We are certain that medical residents are a valued asset for the health care system and a source of high-skill professional that need support while studying their medical specialization. We aimed to provide recommendations to ameliorate some of their mental health problems.

## Limitations

Due to confidentially rules, the research team did not have direct contact with the medical residents, without mediation of the hospital authorities. This indirect contact may had caused under reported scores from participants, even though confidentially was assured.

Another limitation was that the only contact with the medical residents were though email, and the invitation could have missed medical residents whose contact details were wrong or unavailable.

## Supporting information

S1 Table Sociodemographic distribution per residency ranking. The chronic physical illnesses included in this category are diabetes, hypertension, cancer, hypothyroidism, AIDS, etc. Psychological conditions included disorders linked to emotional states, particularly anxiety and depression.
(XLSX)

S2 Table Gender differences in burnout and mental health scores. The degrees of freedom are adjusted by means of the Leven test of homoscedasticity.
(XLSX)

S3 Table Marital status differences in burnout and mental health scores. The degrees of freedom are adjusted by means of the Leven test of homoscedasticity.
(XLSX)

S4 Table Differences between residents with or without physical or psychological illnesses in burnout and mental health scores. The degrees of freedom are adjusted by means of the Leven test of homoscedasticity.
(XLSX)

S5 Table Residency ranking differences in burnout and mental health scores. Post hoc analyses were conducted by means of the Scheffé test.
(XLSX)

S1 Data (SAV)
