# Correction: The moderating role of sociodemographic and work-related variables in burnout and mental health levels of Mexican medical residents

**DOI:** 10.1371/journal.pone.0318611

**Published:** 2025-01-28

**Authors:** Alejandra del Carmen Dominguez-Espinosa, Sandra Irma Montes de Oca-Mayagoitia, Ana Paola Sáenz-Jiménez, Javier de la Fuente-Zepeda, Lilia Monroy Ramírez de Arellano

The third author’s name is spelled incorrectly. The correct name is: Ana Paola Sáenz-Jiménez. The correct citation is: Dominguez-Espinosa AdC, Montes de Oca-Mayagoitia SI, Sáenz-Jiménez AP, de la Fuente-Zepeda J, Monroy Ramírez de Arellano L (2022) The moderating role of sociodemographic and work-related variables in burnout and mental health levels of Mexican medical residents. PLOS ONE 17(9): e0274322. https://doi.org/10.1371/journal.pone.0274322.
